# Determinants of the Relationship between Cytokine Production in Pregnant Women and Their Infants

**DOI:** 10.1371/journal.pone.0007711

**Published:** 2009-11-09

**Authors:** Yenny Djuardi, Heri Wibowo, Taniawati Supali, Iwan Ariawan, Robbert G. M. Bredius, Maria Yazdanbakhsh, Laura C. Rodrigues, Erliyani Sartono

**Affiliations:** 1 Department of Parasitology, Faculty of Medicine, University of Indonesia, Jakarta, Indonesia; 2 Department of Parasitology, Leiden University Medical Center, Leiden, The Netherlands; 3 Department of Population Studies and Biostatistics, School of Public Health, University of Indonesia, Depok, Indonesia; 4 Department of Pediatrics, Bone Marrow Transplant Unit, Leiden University Medical Center, Leiden, The Netherlands; 5 Department of Epidemiology and Public Health, London School of Hygiene & Tropical Medicine, London, United Kingdom; Columbia University, United States of America

## Abstract

Exposure to environmental factors during fetal life and infancy is thought to play an important role in the early development of innate and adaptive immunity. The immunological relationship between mother and infant and the effect that environmental exposures have during pregnancy and early childhood have not been studied extensively. Here the production of cytokines was measured in 146 pairs of mothers and their 2- month-old infants. The effect of place of residence, socio-economic variables, parasitic infections as well as maternal and child characteristics on measured cytokine production was determined. Mothers producing high levels of IL-10, IFN-γ and IL-5 were more likely to have infants who also produced high levels of these cytokines either spontaneously (OR 2.6(95%CI 1.2–5.4), OR 2.9(CI 1.3–6.6), OR 11.2(CI 4.6–27.2), respectively) or in response to PHA (IL-10: OR 3.0(CI 1.4–6.6), IFN-γ: OR 2.0(CI 1.0–4.2), respectively) even after adjustment for potential confounding variables. This was not the case for TNF-α. In response to LPS, place of residence was a strong determinant of infant IL-10 (OR 0.2(CI 0.1–0.9)) and TNF-α (OR 0.3(CI 0.1–0.9)) production. Maternal protozoan infections was independently associated with reduced infant IL10 in response to PHA and to LPS as well as reduced TNF-α and IFN-γ in response to PHA. These results indicate strong relationship between maternal and infant's cellular immune responses even after taking into account many environmental influences that could affect infant's response directly or indirectly through uterine microenvironment. However, place of residence and intestinal infections may still directly affect the immune responses of the infant. Taken together, the study provides evidence for imprinted cytokine responses of an infant which may have implications for their reaction to incoming antigens, warranting further investigation into the role that genetics or epigenetics play in shaping the cytokine response by an infant to self or external antigens.

## Introduction

In utero environment has evolved to ensure that the semi-allogeneic fetus can grow optimally, with placenta as an immunological barrier between maternal and fetal circulation. It is known that maternal nutrient imbalance or exposure to allergens or pathogens may modulate the immune responses of the fetus. The capacity of cord blood mononuclear cells (CBMC) of neonates born to mothers infected with filarial parasite [1]–[3], intestinal helminth [4] or malaria [5], [6] to mount parasite-specific cellular and humoral immune responses is taken as evidence for sensitization of fetal immune cells during the gestational period. The higher CBMC proliferative responses to birch pollen from babies born to mothers exposed to birch pollen during months 5–7 of pregnancy [7], [8] is an indication of early priming to allergens. Furthermore, maternal smoking during pregnancy results in higher cotinine levels in cord blood; this condition is associated with attenuated neonatal innate immune responses and may have an impact on the maturation of antigen presenting cells [9]. In utero exposure to maternal diet such as fish oil supplementation during pregnancy could induce an immunoregulatory effect on infant cytokine production with [10] or without the presence of stimulus such as allergens [11]. Some cross-sectional studies on atopic disorders have shown a correlation between T helper (Th) 1 or Th2 cytokines produced by mothers and their corresponding cord blood cells [12] or produced by their 2 year-old children [13], but the analyses did not consider the role played by environmental factors. It is known that environmental factors can affect fetal life and may have long-term implications for susceptibility or resistance to infections [14], development of metabolic syndromes and cardiovascular diseases [15]–[17], or asthma and allergy [18].

In the present study we have investigated in Indonesia where environmental exposures are highly varied, the relationship between maternal and infant's cellular immune responses at early life before the start of vaccinations. This would circumvent the problems when studying cord blood responses, namely the effect that physiological stress caused during birth might exert and the possible cross contamination with maternal blood. The specific aims of this study were twofold: a) to assess how close the relationship is between cytokine responses in pregnant women and their children and b) to evaluate the associations between environmental factors and maternal characteristics that in turn affect cytokine responses of their children. To this end, a conceptual framework was proposed to define the relationship between environmental factors and maternal characteristics and the infant's immune system. This framework was used to then guide the inclusion of the influential variables in the multiple logistic regression model.

## Methods

### Ethics Statement

This study was conducted according to the principles expressed in the Declaration of Helsinki. The study was approved by Ethics Committee of Faculty of Medicine, University of Indonesia. All mothers were provided written informed consent for the collection of samples from themselves and their children and for subsequent analysis.

### Study Population

The present study was part of a birth cohort study examining the immune responses of children born to helminth infected mothers in Bekasi District, located approximately 30 km from the capital city Jakarta, Indonesia. Between 2002 and 2004, pregnant mothers and their infants were recruited from two adjacent villages, Jati Sampurna (JS) and Jati Karya (JK). These villages are in a peri-urban area, with a mixture of farmers and small traders. All pregnant mothers in second and third trimester from the villages were invited via midwives to participate in the study. Demographic and socio-economic data, as well as maternal characteristics during pregnancy were collected by questionnaires. Gestation age at the time of blood collection was estimated from the last menstrual date and confirmed by palpation and measurement of fundal height. Information about child gender and birth weight was obtained from the mothers during house-to-house visits.

### Parasitological Examination from Maternal Blood and Stool

For determination of microfilaremia, one ml of maternal venous blood collected between 8–11 pm was filtered through 5 µm pore membrane (Millipore, Billerica, MA, USA). Circulating *Wuchereria bancrofti* antigen in maternal blood was detected by using immunochromatographic test (ICT) in a card format (Binax, Scarborough, ME, USA), according to the manufacturer's recommendation. Stool samples were collected and preserved with formalin (10%), then transferred to the laboratory at the Department of Parasitology, University of Indonesia, and examined for the presence of intestinal helminth eggs and protozoan infections.

### Whole Blood Culture

The procedures of whole blood culture are based on optimized protocols developed during pilot studies. Heparinized venous blood obtained from pregnant mothers and their babies was processed within 6 hours after venipuncture. The whole blood was diluted 10 times as described before [19] and was cultured in duplicate, in the presence of phytohaemagglutinin (PHA; 2 µg/ml; Wellcome Diagnostics, Dartford, UK) as the mitogen, lipopolysaccharide (LPS; 100 ng/ml; Sigma-Aldrich chemie, Zwijndrecht, the Netherlands) as an innate immune stimulus, or without stimuli (medium only). The cultures were incubated for 1 day and 6 days in the presence of 5% CO2, at 37°C. The collected supernatants were kept frozen in −20°C until measurement. The concentrations of interleukin (IL)-10 and TNF-α were measured in day 1 supernatant, whereas IL-5, IL-13, IFN-γ were measured in day 6 supernatant. Paired samples of mother and infant were analyzed altogether in the same plate, in order to minimize variation.

### Covalent Coupling of Capture Antibodies to Beads

The beads used to determine cytokine levels were prepared by using reagents described in Table 1. Each of four different capture monoclonal Abs was covalently coupled to four different carboxylated bead sets (Luminexcorp, Austin, TX, USA) as described elsewhere [20], [21]. For each set, 2.5×10^6^ beads were added with PBS buffer to lower the viscosity. The beads were centrifugated at 15000 *g* for 2 min and washed twice with activation buffer (0.1 M NaH_2_PO_4_, pH 6.2), and finally re-suspended in 80 µL activation buffer. *N*-hydroxy-sulfosuccinimide (Sulfo-NHS) and 1-ethyl-3-(3-dimethylaminopropyl)-carbodiimide hydrochloride (EDC) (both from Pierce, Thermo Fischer Scientific, IL, USA) were used to activate the beads. This bead mixture was incubated and shaken for 20 min at room temperature. The activated beads were washed twice with 0.05 M 2-(N-morpholino) ethane sulfonic acid (MES, pH 5.1), added with capture antibodies (50 µg for IL-10, IL-13, TNF-α; and 100 µg for IFN-γ) and incubated for 2 hrs. The beads were washed twice with PBS/0.5% Tween 20 and were re-suspended in PBS with 10 mg/ml BSA and 0.05% sodium azide, then added with 100 µl 20% sucrose. Finally, the beads were counted using a hemocytometer to get a concentration of 10^6^ per ml. Goat F(ab')2 anti-mouse Ig conjugated to R-phycoerythrin (Southern Biotech, Birmingham, Alabama, USA) was used to estimate the density of the monoclonal Abs coupled to the beads.

**Table 1:** Recombinant proteins and antibodies used in multiple bead-based assay*.

Recombinant protein			Capture Ab			Detection Ab		
Cytokine	Cat. No.	Source	Clone	Cat. No.	Source	Clone	Cat. No.	Source
IL-10	M191003	Sanquin	IL10-5	M9210	Sanquin	IL10-2	M9216	Sanquin
TNF-α	PHC3015	BS	TNFα-7	M9179	Sanquin	TNFα-5	M9218	Sanquin
IL-13	94/622	NIBSC	IL13-1	M9186	Sanquin	IL13-2	M9217	Sanquin
IFN-γ	PHC4031	BS	MD5	M9159	Sanquin	MD2	M9219	Sanquin

*All reagents listed were obtained from the sources indicated from the following abbreviations: Sanquin  =  Stichting Sanquin Bloedvoorziening (Amsterdam, The Netherlands); BS  =  BioSource (Nivelles, Belgium); NIBSC  =  National Institute for Biological Standards & Controls (Potters Bar, UK), with catalogue numbers (Cat. No.) for each reagent given.

### Determination of Cytokine Production by Multiplex Bead-Based Assay

The optimizations of multiple bead-based assay and the cytokine measurements were performed as described [21]. Briefly, single and multiple bead based assays were performed to determine the optimal concentration of the detection antibody, incubation times and reporter signal. From these assays, a bead mixture of IL-10 and TNF-α for day 1 supernatant, IL-13 and IFN-γ for day 6 supernatant were generated freshly before use and mixed with biotinylated detection antibodies (IL-10, 500 ng/ml; TNF-α, 500 ng/ml; IL-13, 25 ng/ml, IFN-γ, 100 ng/ml). Standard curves from each recombinant protein were prepared from three-fold dilution steps in HPE buffer (CLB Sanquin, Amsterdam, The Netherlands) supplemented with 2% sucrose (HPE/S). Samples (diluted twice with HPE/S) and standards in a final volume of 40 µl per well were placed in a 96-well round-bottomed microplates (Nunc, Roskilde, Denmark). Next, 10 µl of the mixture of beads was added to each well and incubated under continuous shaking overnight in the dark. Beads were washed twice with PBS/0.05% Tween 20. The reporter signal, streptavidine PE (Becton Dickinson, San Jose, CA, USA), was added and the bead mixture was incubated for 30 min under continuous shaking. Before reading, the beads were washed once with PBS/0.05% Tween 20 and were reconstituted in a final volume of 70 µl HPE/S.

Mean fluorescent intensity from all cytokines was measured using Luminex IS 100 (Luminexcorp, Austin, TX, USA) and data were analyzed by Star Station software analysis (Applied Cytometry, Sheffield, UK). The measurements were done once, and blank values were substracted from all readings. The minimum detection limit was determined by adding two standard deviations to the mean of mean fluorescence intensity from 30 blanks assayed separately. The detection limits for IL-10, TNF-α, IL-13 and IFN-γ were 6.5 pg/ml, 1.7 pg/ml, 12.5 pg/ml, and 3.6 pg/ml, respectively.

### IL-5 ELISA

IL-5 was measured by ELISA as described previously [22]. Matched antibody pairs, consisting of purified rat anti-mouse/human IL-5 monoclonal antibodies and biotinylated rat anti-human IL-5 monoclonal antibodies were purchased from Becton Dickinson Biosciences Pharmingen, San Jose, CA, USA. Recombinant IL-5 protein was used as standard (Genzyme, Cambridge, UK). The detection limit for IL-5 ELISA was 2 pg/ml.

### Statistical Analyses and Conceptual Framework

All cytokine levels below detection limit were given half of the threshold value. Raw cytokine productions were used for analysis, since the results showed the cytokine responses to antigen stimulation not only higher or the same, but also lower than spontaneous cytokine productions.

Mothers were classified into high producers (H) or low producers (L) based on median cytokine levels. Since almost all cytokine data from mothers and infants were not normally distributed, the Mann-Whitney *U*-test was used to compare levels of cytokine production in infants born to high or low producer mothers. Pearson Chi-Square test was used to find association between two dichotomous variables such as between place of residence and cytokine producer status or between maternal education and the use of cooking fuel.

We used multivariable logistic regression model to investigate the association between mother's cytokine production and infant's cytokine production. The outcome for logistic regression model was infant's cytokine which was grouped into: high producer and low producer, based on the median. Mother's cytokine production was treated as exposure variable. Other variables, such as demographic and socio-economic factors, maternal characteristics, maternal parasitological data and child characteristics, were treated as potential confounders.

**Figure 1:**
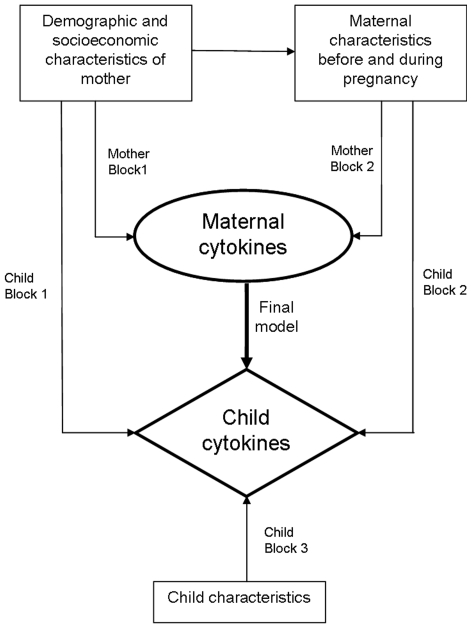
Conceptual framework for logistic regression analysis of the relationships between maternal and infant cytokine responses. Mother Block 1 consists of univariate and multivariate logistic regression models for maternal demographic and socio-economic data, such as place of residence, nativity, education, material of house, water supply, cooking fuel. The outcome variable is maternal cytokine producer status. Mother Block 2 consists of univariate and multivariate logistic regression models for maternal characteristics, such as number of age, number of pregnancies, parasitological data. The outcome variable is maternal cytokine producer status. Child Block 1 consists of univariate and multivariate logistic regression models for maternal demographic and socio-economic data, such as place of residence, nativity, education, material of house, water supply, cooking fuel. The outcome variable is child cytokine producer status. Child Block 2 consists of univariate and multivariate logistic regression models for maternal characteristics, such as number of age, number of pregnancies, parasitological data. The outcome variable is child cytokine producer status. Child Block 3 consists of univariate and multivariate logistic regression models for child characteristics, such as birth weight, mode of delivery, breast feeding. The outcome variable is child cytokine producer status.

The original plan for the logistic regressions was based on a conceptual framework (Figure 1) of the proposed causal pathways [23], [24]. Since maternal – infant immune relationships is the central question of this analysis, we initially performed a simple logistic regression analysis to obtain crude odds ratios (ORs) of the effect of level of each cytokine production in the mother on the level of the same cytokine production on the infant. We then selected potential confounding variables by identifying environmental factors and maternal characteristics that were associated with level of cytokine production in both pregnant mothers and their offspring. A variable was said to be a potential confounder if the introduction of that variable into the model lead to a change in the OR of the association between maternal cytokine and child cytokine of more than 10 percent. Besides confounding effect of other variables, we also tested for interaction between variables.

Environmental factors included were demographic and socio-economic variables (Table 2): material of house (categorized into two groups with semi wood/brick was added to wood as the reference); water supply (pipe users were included in the group of pump users and well users were used as the reference). Maternal characteristics during pregnancy included were age (above or below the mean), number of pregnancies, gestational age at the time of blood collection and maternal parasitological status (two groupings, based on circulating filarial antigen/ICT positivity and on intestinal parasitological status, i.e. the presence of intestinal helminth and/or intestinal protozoan infection). Selection of potential covariates for logistic regression with the binary outcome producer status of mother was through 2 blocks of separated logistic regression analysis, which were of demographic and socioeconomic characteristics of mother, maternal characteristics before and during pregnancy. In each block, variables with p value less than 0.25 were treated as covariates and a multivariable logistic regression was done with all potential covariates. All variables with p value less than 0.05 in multiple logistic regression for potential covariates in each block were included for the next analysis. A final logistic regression analysis was done with all covariates which showed p value less than 0.05 in the previous analysis. As the final model, only variables with p value less than 0.05 were retained in the model.

**Table 2:** Characteristics of pregnant mothers and their infants included in the logistic regression models.

Demographic and socio-economic data		Maternal parasitological data	
**Maternal residence**		**Filarial infection**	
Jati Sampurna village	86/170 (51%)	Microfilaria positive	8/170 (5%)
Jati Karya village	84/170 (49%)	ICT positive	41/170 (24%)
Native	119/169 (70%)	**IH infection**	
Non-native	50/169 (30%)	*Ascaris lumbricoides*	24/161 (15%)
**Maternal education**		*Trichuris trichiura*	12/161 (7%)
Never schooled or primary school	113/169 (67%)	Hookworm	28/161 (17%)
Higher education	56/169 (33%)	**Any IH infection**	57/161 (35%)
**Maternal occupation**		**Any helminth infection** *	79/161 (49%)
Unemployed	156/169 (92%)	**IP infection**	
Others (trader, employee)	13/169 (8%)	*Blastocystis hominis*	36/161 (22%)
**Material of house**		*Entamoeba histolytica/dispar*	6/161 (4%)
Wood	44/169 (26%)	*Endolimax nana*	5/161 (3%)
Brick	121/169 (72%)	*Entamoeba coli*	1/161 (1%)
Semi wood/brick	4/169 (2%)	*Iodamoeba butschlii*	2/161 (1%)
**Water supply**		**Any IP infection**	44/161 (27%)
Well	47/169 (28%)	**Status of IH and IP infections**	
Pump	120/169 (71%)	No IH or IP infection	82/161 (51%)
Pipe	2/169 (1%)	IH infection only	35/161 (21%)
**Cooking fuel**		IP infection only	22/161 (14%)
Wood	22/167 (13%)	Co-infection of IH and IP	22/161 (14%)
Kerosene	125/167 (75%)		
Gas	20/167 (12%)		
**Maternal characteristics**			
**Number of pregnancies**			
Primigravid	56/169 (34%)		
Multigravid	113/169 (66%)		
**Mean maternal age, years (SD)**	25.5 (5.9)		
< 25 yrs	84/169 (50%)		
≥25 yrs	85/169 (50%)		
**Child characteristics**			
**Median birth weight, g (IQR)**	3200 (3000–3500)		
**Mode of delivery**			
Vaginal	138/142 (97%)		
Caesarian section	4/142 (3%)		
**Breast feeding, months**			
Exclusive breast feeding	101/119 (85%)		
Partial breast feeding	13/119 (11%)		
No breast feeding	5/119 (4%)		

*any helminth infection: either single or mixed infections of intestinal helminth and filarial.

IH  =  Intestinal helminth, IP  =  Intestinal protozoan.

The same steps were applied to identify the variables which influenced the infant production of cytokines, logistic regressions with the binary outcome producer status of the child, and exposures were grouped into the two previous blocks, environmental factors and maternal characteristics during pregnancy and a third block of child characteristics: gender, birth weight, mode of delivery and breast feeding status: exclusive breast-feeding (receiving only breast milk for at least 6 months), partial breast-feeding (receiving breast and formula milk), or no breast-feeding. The variables from first and second child blocks were considered as more distal determinants than the third child block [24], so the selected variables from child block 1 and 2 were modeled together and later on the selected variables from this model were added to the selected variables from child block 3 in a new regression model. A final model for infant's cytokine was created with maternal cytokine producer status and confounding factors for maternal and child cytokine production. In this paper we will present only the table of crude OR for maternal cytokines and the table of adjusted OR in final model for maternal cytokines and potential confounding factors. Additional results (other than the two tables presented here) are available from the corresponding author. Information on child gender and age at the time of blood collection was collected but not included in the models since these child characteristics had no influence on maternal cytokines and maternal-infant cytokine relationships. Gestational age at time of blood collection which might have influence on maternal cytokines but not on child cytokines was not included in the models.

All statistical analyses were performed using SPSS version 15. Hosmer-Lemeshow Goodness-of-Fit test was done at final step for each cytokine/stimuli, to ensure that the final model adequately fit the data.

## Results

### Study Subjects

One hundred and seventy mothers in second and third trimester of pregnancy donated their blood for immunological studies and subsequently after birth one hundred and forty six infants between 1 to 17 weeks old (before any vaccination) participated in the study. Twenty four infants could not be included in the study due to refusal of parents to donate their infant blood or due to infant death, being sick, moving outside the study area, or being untraceable. The analysis of maternal and infant relationships was done for 146 pairs of mother and child for spontaneous or mitogen-induced cytokine production, and 74 pairs of mother and child for LPS-induced cytokine production. The reason for lower number for LPS is the late arrival of this stimulus, at a time point when the study has already started.

Table 2 shows the characteristics of the study population and includes the demographic and socioeconomic details along with the pregnancy and infection status of the mothers as well as the relevant child data. The median age of the infants at the time of blood collection was 4.6 weeks (IQR = 2.1–8.2 weeks) and the proportion of girls was 51%. For pregnant women, the median gestational age at the time of blood collection was 28 weeks (IQR = 24–32 weeks) with 60% of samples collected in the third trimester and the rest in the second trimester of pregnancy. Most births (97%) were vaginal delivery and most infants (85%) were breastfed. The majority of the mothers (67%) had a low education level. The water sources in 71% of the study population were from hand pumps, 28% from wells. Since there was no data about the water sanitation, we were not able to compare which of these two water sources was considered to be more hygienic. Maternal filarial infection as determined by circulating antigen was 24% while 35% and 27% of mothers were infected with intestinal helminths and protozoa, respectively.

### Relationship between Maternal and Infant Cytokine Responses

**Figure 2:**
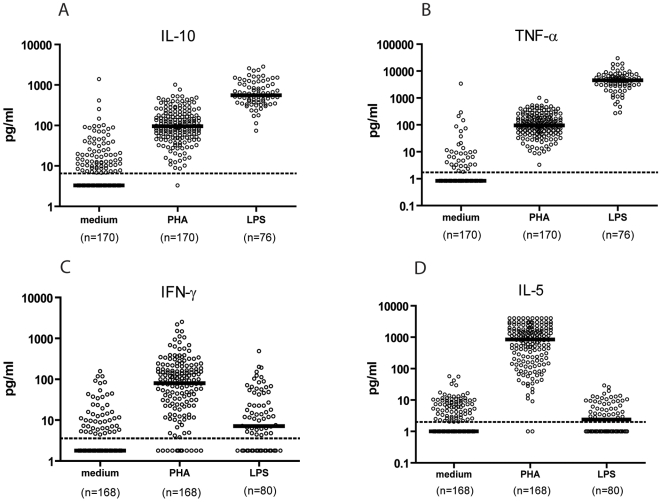
Maternal cytokine production. Solid lines represent median levels of each cytokine; broken lines represent the detection limits of each cytokine. Each dot represents one individual. The number of non-detectables are given in parenthesis: (A) IL-10 medium, PHA, LPS (93, 1, 0); (B) TNF-α medium, PHA, LPS (135, 6, 0); (C) IFN-γ medium, PHA, LPS (119, 22, 28); (D) IL-5 medium, PHA, LPS (91, 2, 37).

Maternal cytokine responses, spontaneous (to medium), to PHA and to LPS are given in Figure 2. The pattern of maternal IL-13 production in response to various stimuli was similar to IL-5 (data not shown). As indicated in Methods, the median cytokine production was used to stratify mothers into high and low cytokine producers.

**Figure 3:**
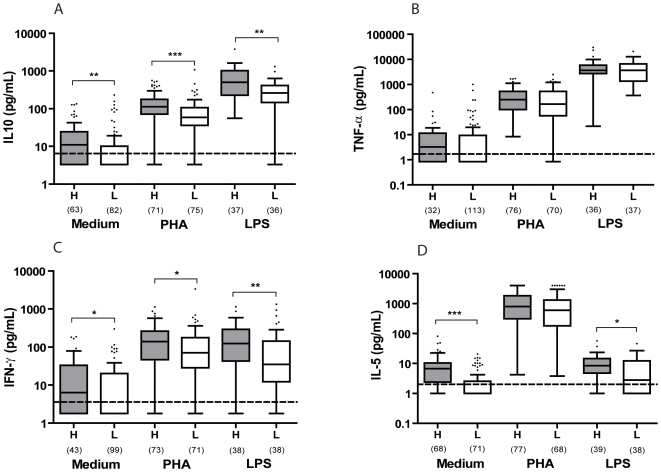
Comparisons of cytokines in infants born to High producer (H) or to Low producer (L) mothers. A: IL-10, B: TNF-α, C: IFN-γ, D: IL-5. The line within the box represents the median (50^th^ percentile), with the lower and upper borders representing the interquartile range (25^th^ and 75^th^ percentiles). The whiskers extend to the 10^th^ and 90^th^ percentiles. The closed dots represent values above the 90^th^ percentiles. Detection limit of each cytokine is shown as a broken line. *0.05>*p*≥0.01; **0.01>*p*≥0.001; ***0.001>*p*.

As a whole, the comparison between cytokine levels of infants born to high and low producer mothers revealed that infants born to high producer mothers had significantly higher IL-10, IL-5 and IFN-γ responses (Figure 3). This was true either for spontaneous or LPS stimulated cytokines. Although TNF-α responses showed a similar trend, the difference between infants born to mothers with a high or a low TNF-α production was not statistically significant. Similarly, we found IL-10 and IFN-γ to PHA was higher in infants born to high producer mothers compared to those born to low producer mothers.

Table 3 shows the increase in likelihood of a child being a high producer of each cytokine either spontaneously or in response to PHA and LPS when the mother is a high producer of the corresponding cytokines.

**Table 3:** Crude odds ratios for high cytokine production by a child according to maternal cytokine production.

Maternal Cytokine	Medium		PHA		LPS	
	Crude OR (95% CI)	*p-*value	Crude OR (95% CI)	*p-*value	Crude OR (95% CI)	*p-*value
IL-10						
Low producers	reference		reference		reference	
High producers	2.9 (1.5–5.8)	0.002	4.2 (2.1–8.3)	<0.001	3.3 (1.3–8.5)	0.02
TNF-α						
Low producers	reference		reference		reference	
High producers	1.8 (0.8–4.0)	0.14	1.7 (0.9–3.4)	0.10	0.7 (0.3–1.8)	0.49
IFN-γ						
Low producers	reference		reference		reference	
High producers	2.6 (1.2–5.4)	0.01	1.9 (1.0–3.7)	0.06	3.0 (1.2–7.8)	0.02
IL-5						
Low producers	reference		reference		reference	
High producers	8.4 (3.9–17.9)	<0.001	1.7 (0.9–3.3)	0.11	2.7 (1.1–6.9)	0.03

### Model for Infant's Cytokine Production

Table 4 summarizes final logistic regression model for each cytokine after including maternal cytokine producer status, potential confounding factors from block 1 and 2 of maternal cytokines and potential confounding factors from block 1, 2 and 3 of child cytokines (see Methods). The findings for each cytokine are given below:

**Table 4:** Multivariable analysis giving adjusted odds ratios for high cytokine response of a child according to maternal cytokine responder status and maternal characteristics.

	Medium		PHA		LPS	
	Adjusted OR (95% CI)	*p-*value	Adjusted OR (95% CI)	*p-*value	Adjusted OR (95% CI)	*p-*value
Maternal IL-10						
Low producers	reference		reference		reference	
High producers	2.6 (1.2–5.4)	**0.01**	3.0 (1.4–6.6)	**0.005**	1.6 (0.5–5.9)	0.45
Village						
JS			reference		reference	
JK			0.7 (0.3–1.5)	0.38	0.2 (0.1–0.9)	**0.03**
Education						
Low			reference			
High			0.7 (0.3–1.7)	0.44		
Cooking fuel						
Wood	reference					
Kerosene	1.3 (0.4–3.7)	0.68				
Gas	0.5 (0.1–2.3)	0.34				
Status of IH and IP infections						
Negative	reference		reference		reference	
IH only	0.5 (0.2–1.4)	0.20	0.7 (0.3–1.7)	0.39	0.5 (0.1–2.3)	0.38
IP only	0.5 (0.2–1.4)	0.47	0.3 (0.1–0.9)	**0.04**	0.1 (0.03–0.6)	**0.01**
IH + IP	0.8 (0.3–2.3)	0.71	0.5 (0.2–1.7)	0.29	0.2 (0.1–0.9)	**0.03**
Maternal TNF-α						
Low producers	reference		reference		reference	
High producers	1.7 (0.7–4.1)	0.20	1.9 (0.9–4.0)	0.11	0.5 (0.2–1.5)	0.23
Village						
JS			reference		reference	
JK			1.7 (0.8–3.8)	0.16	0.3 (0.1–0.9)	**0.03**
Native			reference			
Non native			1.9 (0.8–4.5)	0.17		
Education						
Low	reference		reference			
High	0.4 (0.2–0.9)	**0.04**	0.7 (0.3–1.7)	0.44		
Cooking fuel						
Wood	reference					
Kerosene	2.6 (0.8–7.8)	0.10				
Gas	17.1 (3.0–98.1)	**0.001**				
Status of IH and IP infections						
Negative			reference			
IH only			1.3 (0.5–3.4)	0.54		
IP only			0.2 (0.04–0.6)	**0.008**		
IH + IP			0.7 (0.2–2.1)	0.56		
Maternal IFN-γ						
Low producers	reference		reference		reference	
High producers	2.9 (1.3–6.6)	**0.01**	2.0 (1.0–4.2)	**0.05**	2.8 (1.03–7.8)	**0.04**
Village						
JS	reference				reference	
JK	0.4 (0.2–0.9)	**0.02**			0.2 (0.1–0.6)	**0.003**
Education						
Low	reference					
High	0.4 (0.2–0.9)	**0.03**				
Occupation						
Unemployed			reference			
Others			0.7 (0.2–2.8)	0.62		
No. of pregnancies						
Primigravid	reference					
Multigravid	0.5 (0.2–1.2)	0.11				
Status of IH and IP infections						
Negative			reference			
IH only			0.9 (0.4–2.1)	0.79		
IP only			0.2 (0.1–0.7)	**0.01**		
IH + IP			0.6 (0.2–1.7)	0.37		
Maternal IL-5						
Low producers	reference		reference			
High producers	11.2 (4.6–27.2)	**<0.001**	1.7 (0.8–3.3)	0.15		
Village						
JS	reference					
JK	1.3 (0.6–2.9)	0.51				
Maternal age						
<25 yrs	reference					
≥25 yrs	2.8 (1.2–6.9)	**0.02**				
Status of IH and IP infections						
Negative			reference			
IH only			2.2 (0.9–5.3)	0.08		
IP only			1.2 (0.4–3.2)	0.76		
IH + IP			1.5 (0.5–4.1)	0.43		

OR  =  Odds ratio; **Bold**  =  significant association.

JS  =  Jati Sampurna, JK  =  Jati Karya, IH  =  Intestinal helminth, IP  =  Intestinal protozoan.

#### Model for infant IL-10 production

Mothers with higher spontaneous IL-10 production had children with higher spontaneous IL-10 release (OR 2.6(95%CI 1.2–5.4)) (Table 4). The value is very similar before and after controlling for environmental measures, indicating that none of the factors examined confounded the relationship between mother and child on spontaneous IL-10.

Mothers with high IL-10 in response to PHA had children with high IL-10 in response to PHA (OR 3.0(95%CI 1.4–6.6)), but the value is much lower than before controlling for environmental measures; this was most marked when controlling for maternal intestinal protozoa, indicating that intestinal protozoan infection is an independent predictor of a child's IL-10 production in response to PHA and a confounder of this relationship. Village of residence and maternal education, which were associated with high IL-10 production of a child in response to PHA were no longer significant after adjustment for mother's IL-10 production against PHA.

Mothers with high IL-10 in response to LPS had children with high IL-10 in response to LPS, but the magnitude of association was much smaller and no longer significant when maternal intestinal parasitic infections and village of residence were adjusted for. Both mothers (Chi-Square test, *p*<0.001) and infants born to mothers from JK village had lower IL-10 in response to LPS (OR 0.2(95%CI 0.1–0.9)). Since there is biological plausibility for place of residence influencing level of IL-10 production but level of IL-10 production can not influence place of residence then the direction of this association must be place of residence causing levels of IL-10 production rather then the other way round. Since the relationship between levels of production between mother and child disappears when village is controlled for, the only plausible explanation is that village of residence was influencing both maternal and child cytokine levels. Having intestinal protozoan infection alone or mixed with intestinal helminths was independently associated with lower levels of infant's IL-10 production in response to LPS (OR 0.1(95%CI 0.03–0.6), OR 0.2(95%CI 0.1–0.9), respectively).

#### Model for infant TNF-α production

There were no significant associations between maternal and infant TNF-α production (Table 4). However, several maternal factors such as education and cooking fuel had significant direct effect on infant spontaneous TNF-α production. Higher education of mother was associated with lower spontaneous TNF-α production by her child (OR 0.4 (95%CI 0.2–0.9)). Using gas (OR 17.1(95%CI 3.0–98.1)) and kerosene (OR 2.6(95%CI 0.8–7.8)) as cooking fuel was positively associated with spontaneous TNF-α release in children. Mothers with higher educational levels were more likely to cook using gas stove than wood (Chi-Square test, *p*<0.001).

Intestinal protozoan infection of mothers was significantly associated with lower TNF-α responses to mitogen in infants (OR 0.2(95%CI 0.04–0.6)). Other variables were not significant anymore after adjustment. TNF-α production of infant in response to LPS was not associated with any of maternal factors, except for residence, where infants born and living in JK had significantly lower levels than those born in JS (OR 0.3(95%CI 0.1–0.9)).

#### Model for infant IFN-γ production

Maternal spontaneous IFN-γ response was significantly associated with child cytokine response (OR 2.9(95%CI 1.3–6.6)), after adjustment for residence (OR 0.4(95%CI 0.2–0.9)) and educational level (OR 0.4(95%CI 0.2–0.9)). Residence factor increased the crude OR for maternal-infant relationship in spontaneous IFN-γ by 19% (adjusted OR 3.1(95%CI 1.4–6.8)). As seen for maternal IL-10 response to LPS, maternal spontaneous IFN-γ production was the mediator between residence factor and infant spontaneous IFN-γ. Number of pregnancies, which was significantly associated with maternal IFN-γ production, lost significance in the final child model.

The relationship between maternal and child's IFN-γ was less significant in response to PHA (OR 2.0(95%CI 1.0–4.2)). Maternal intestinal protozoan infection had a stronger effect on child cytokine production (OR 0.2(95%CI 0.1–0.7)) than maternal IFN-γ. In responses to LPS, maternal IFN-γ production was a significant determinant of the infant's IFN-γ production (OR 2.8(95%CI 1.0–7.8)) although the effect of residence was stronger (OR 0.2(95%CI 0.1–0.6)). Since the level of IFN-γ production in response to LPS was considered low in mothers and infants (Figure 2, 3), this maternal-infant association could partly reflect the association found in the production of spontaneous IFN-γ. Indeed, the association between maternal and infant IFN-γ was no longer significant when net LPS-stimulated IFN-γ (spontaneous IFN-γ subtracted from LPS-stimulated IFN-γ) by mothers and infants was used in the regression model (data not shown).

#### Model for infant's IL-5 production

For spontaneous IL-5 release, there was a significant association between maternal and infant responses (OR 11.2(95%CI 4.6–27.2)). Younger age of mother was associated with higher spontaneous IL-5 production (data not shown) but not with infant's IL-5; however in the final model (Table 4) including maternal age lead to changes in the the maternal-infant relationship. The crude OR for maternal-child spontaneous IL-5 (Table 3) increased by 39% when adjusted for maternal age (OR 11.7(95%CI 4.9–28.1)), showing that maternal age had an indirect effect on infant IL-5 through maternal IL-5 as the mediator. Maternal residence was no longer significantly associated with infant's spontaneous IL-5 after adjustment with maternal cytokine and maternal age.

Maternal IL-5 responses to PHA had no significant effect on child's IL-5 to PHA, however there was a tendency for intestinal helminth infections of mother to be associated with higher IL-5 responses to PHA of infants (OR 2.2(95%CI 0.9–5.3)).

## Discussion

This study indicates that maternal cytokine responses are important determinants of the corresponding cytokines in infants during early life. This was particularly the case for spontaneous production of cytokines. Spontaneous production of IL-10, IFN-γ and IL-5 by two month old infants was strongly determined by maternal cytokine and was not influenced by any other environmental variables recorded in our study. Relationship between maternal cytokine responses to PHA and the corresponding infant's cytokine production was also found in the production of IL-10, and to lesser extent of IFN-γ. The findings, especially for IL-10 production, that infants up to 17 weeks still inherited a similar intrinsic capacity to produce this cytokine as their mothers is supported by the findings of a cross-sectional study of allergic and non allergic mothers in Europe, showing that the production of IL-10 and IFN-γ in response to medium (spontaneous production) and after PHA stimulation were correlated between mothers and their 2 year-old children irrespective of maternal atopic status [13] and measured environmental factors such as month of birth, length of breastfeeding, smoking parents, having pets at home, number of sibling, and day care attendance. We show that area of residence is a strong confounder for infant IL-10 and TNF-α response to LPS. LPS as a Toll-like receptor 4 ligand and a major component of Gram-negative bacterial cell wall is a strong stimulus for innate immune responses. The lower responses to LPS in JK infants was interesting as the JK village had higher prevalence of intestinal parasite infections (data not shown) which would suggest lower standards of hygiene and in turn higher chance of exposure to bacterial pathogens. It is known that continuous exposure to high microbial or parasitic stimuli may result in down-regulation of the TLR function as shown by some *in vitro* studies with human epithelial cell lines [25], [26] or in studies of school children living in rural areas of some European [27] or African [28] countries. Interestingly, comparison of TLR expression on cells of the immune system between urban European neonates and Gabonese neonates who in a semi urban area are exposed to high burden of infections, indicated a significantly lower expression TLR-2 on Gabonese cells, suggesting that there is a very early down regulation of TLRs possibly as a result of in utero exposure to micro organisms and parasites [29]. We noted that maternal IL-10 and TNF-α production in response to LPS (during pregnancy) was lower in JK compared to JS (data not shown) as was those of their infants. Indeed the relationship between maternal and infant responses to LPS was primarily accounted for by place of residence. We also realized that the environmental factors included in the analysis of our study may not be complete, and residence may represent several environmental parameters not measured in our study such as exposure to pets or livestock, maternal nutritional status or access to sanitation before and during pregnancy. With respect to the latter a recent study in Brazil, found high spontaneous IL-10 responses in children without access to safe drinking water or sewage system [30].

TNF- α as a pro-inflammatory cytokine was shown to have no strong associations between mother and child. This may simply suggest that the environment in the first several months of an infant's life has a strong effect on the immune system. For example, infections such as rotavirus which are prevalent very early in infants may alter the TNF-α responses [31]. However, in the case of spontaneous TNF-α, maternal education and cooking fuel seem to independently affect infant cytokine responses.

We used crosstabs to find the association between education and the use of cooking fuel. The result showed that higher education may be associated with higher economic status, which explains why this group used more gas stove than wood. The finding that cooking fuel only affected the child's spontaneous TNF-α release but not maternal cytokine may indicate that child immune system is more vulnerable to this kind of environmental exposure.

With respect to maternal infections, the final model of multiple logistic regression in our study showed that intestinal parasitic infections especially protozoa influenced the relationship between maternal and infant IL-10 and IFN-γ production in responses to PHA. *Blastocystis hominis* was the most prevalent species of protozoa found in our pregnant subjects. The presence of *B. hominis* could be an indicator for environmental contamination [32]. However as the effect was on PHA and unlike the village effect which was on LPS stimulated cytokine responses, the protozoa may be affecting adaptive rather than innate immune responses. To our knowledge this is the first study that has found an interaction between intestinal protozoan infection and cytokine production in pregnant mothers and their infants which needs to be studied further.

In conclusion this study provides evidence for strong associations between maternal and infant cytokine responses in geographical areas where environmental exposures are highly varied such as in Indonesia. However, the mechanisms behind the strong associations have not been elucidated and form the basis for future studies. It is possible that maternal cytokine responses specifically drive the infant cytokine responses, either by crossing or transmitting signals through the maternal-fetal interface. There is so far no evidence for such direct cross talk between mother and fetus. It is also possible that as yet unidentified environmental factor affects both maternal and infant cytokine responses leading to the correlations observed. Another possibility lies in the genetic link between mother and infant. Whether such cytokine imprinting affects infant's responses to vaccinations or incoming infections needs to be studied in longitudinal manner along with possible associated genetic or epigenetic modifications.
